# Changes in hematological, biochemical, and blood gases parameters in response to progressive inclusion of nitrate in the diet of Holstein calves

**DOI:** 10.14202/vetworld.2021.61-69

**Published:** 2021-01-09

**Authors:** Abimael Ortiz-Chura, Gisela Marcoppido, José Gere, Gustavo Depetris, Francisco Stefañuk, Marcos D. Trangoni, Silvio L. Cravero, Claudia Faverín, Angel Cataldi, María E. Cerón-Cucchi

**Affiliations:** 1 Institute of Pathobiology, National Institute of Agricultural Technology, National Scientific and Technical Research Council, Hurlingham (C1686), Argentina; 2 Engineering Research and Development Division, National Technological University, National Scientific and Technical Research Council, Ciudad Autónoma de Buenos Aires (C1179), Argentina; 3 Agricultural Experimental Station of Balcarce, National Institute of Agricultural Technology, Balcarce (B7620), Argentina; 4 Institute of Agrobiotechnology and Molecular Biology, National Institute of Agricultural Technology, National Scientific and Technical Research Council, Hurlingham (C1686), Argentina

**Keywords:** dry matter intake, liver function, methemoglobin, nitrate toxicity

## Abstract

**Background and Aim:**

Nitrate (NO_3_^-^) reduces enteric methane emissions and could be a source of non-protein nitrogen in ruminant feeds. Nonetheless, it has a potential toxic effect that could compromise animal health and production. The purpose of this study was to determine the effects of progressive inclusion of NO_3_^-^ in the diet on the hematological, biochemical, and blood gases parameters, in turn, the effects on feed intake and live weight gain (LWG) in Holstein calves.

**Materials and Methods:**

Eighteen Holstein heifers and steers (nine animals/treatment) were maintained in individual pens for 45 days. Animals were randomly allocated to either a control or nitrate diet (ND) (containing 15 g of NO_3_^-^/kg of dry matter [DM]). The biochemical parameters and blood gases were analyzed only in the NO_3_^-^ group on days: -1, 1, 7, 13, 19, and 25 corresponding to 0, 20, 40, 60, 80, and 100% of the total inclusion of NO_3_^-^ in the diet, respectively. In addition, DM intake (DMI) and LWG were evaluated among dietary treatments.

**Results:**

Feeding the ND did not influence DMI or LWG (p>0.05). Methemoglobin (MetHb) and deoxyhemoglobin increased according to the NO_3_^-^ concentrations in the diet (p<0.05), while an opposite effect was observed for oxyhemoglobin and carboxyhemoglobin (p<0.05). Hematocrit levels decreased (p<0.05), while albumin, alanine aminotransferase, and gamma-glutamyl transpeptidase concentrations were not modified (p>0.05). However, glucose, urea, aspartate aminotransferase (AST), and retinol concentrations increased (p<0.05) according to the NO_3_^-^ concentrations in the diet.

**Conclusion:**

This study confirmed that the progressive inclusion of 123 g of NO_3_^-^/animal/day in the diet could be safe without affecting DMI and LWG of Holstein calves. In turn, a dose-response effect of the MetHb, glucose, urea, AST, and retinol was observed, but these values did not exceed reference values. These results highlighted the importance of using a scheme of progressive inclusion of NO_3_^-^ in the diet of calves to reduce the risks of NO_3_^-^ toxicity.

## Introduction

The use of nitrate (NO_3_^-^) in ruminants’ diet decreases enteric methane (CH_4_) emissions, which tested in *in vitro* and *in vivo* studies, showing effective and persistent results as an option in methane mitigation [1]. This reduction relies on that NO_3_^-^ consumes more electrons at the expense of CH_4_ production, by reducing it to nitrite (NO2_-_) and also to ruminal ammonia (NH_3_) [2]. In this sense, the presence of NO_3_^-^ in the rumen drives a shift in the use of hydrogen (H_2_) toward NH_3_ production instead of CH_4_ production [2]. However, when the input of NO_3_^-^ exceeds the ruminal microbiota ability for NO_3_^-^ reduction, this mechanism is altered, causing the NO2_-_ to accumulate in the rumen and pass into the bloodstream, resulting in increased methemoglobinemia in ruminants [3]. Signs of NO_3_^-^ toxicity may appear when more than 20% of the hemoglobin is converted to methemoglobin (MetHb) [4]. Symptoms depend on the degree of exposure to NO_3_^-^, such as decreased feed intake resulting in reduced live weight gain (LWG), susceptibility to infections, reproductive inefficiency, brown mucous membrane discoloration, respiratory distress, coma, cyanosis, and even death [5].

A key condition for the use of NO_3_^-^ or other additives as anti-methanogenic agents is that they do not develop harmful effects on animal health and performance [6]. Several strategies have been developed to reduce the risks of toxicity by NO_3_^-^ inclusion in the diet, such as NO_3_^-^ encapsulation [7] and forages sprayed with NO_3_^-^ [8]. In addition, the gradual adaptation to NO_3_^-^ in the diet could be an alternative to minimize negative effects. The previous study have shown that progressive inclusion of NO_3_^-^ in the diet did not compromise animal performance, produced no toxic effects, and had no cumulative effects on the animal products [9]. However, these previous studies were focused only on assessing the effects on the abatement capacity of enteric CH_4_ emissions, and monitoring of blood MetHb as the unique indicator of NO_3_^-^ toxicity [10,11]. In this sense, many studies have often ignored hematological, biochemical, and blood gases changes during the adaptation period to dietary NO_3_^-^, although the importance of the inclusion of these compounds on animal health.

The mitigation potential of NO_3_^-^ can not only be beneficial in intensive milk and meat production systems but can also be especially interesting in pasture-based livestock systems that use low protein forages to maintain animal production (mainly during the dry season), because the ruminal microbiota of the host animal can benefit from NO_3_^-^ as a non-protein nitrogen source and use it for microbial protein synthesis. Therefore, the use of NO_3_^-^ would not only reduce the environmental impact but also improve animal performance, such as was evidenced in the study by Wang *et al*. [12].

Our study highlights the importance of the adaptation period and animal response to NO_3_^-^ in the diet. We hypothesize that the progressive inclusion of NO_3_^-^ in the diet allows an effective adaptation of the NO_3_^-^ reducing ruminal microbiota, which causes a dose-response effect on hematological, biochemical, and blood gases parameters without reaching toxicity levels for the animal, and without causing changes in animal performance. Thus, the aim of this study was to evaluate the effects of progressive inclusion of NO_3_^-^ in the diet on the hematological, biochemical, and blood gases parameters, in turn, the effects on feed intake and LWG in Holstein calves.

## Materials and Methods

### Ethical approval

This study was performed in accordance with international recommendations specified in the guidelines for the use and care of animals. All the animal procedures used in this study were approved by the Committee for Use and Care of Experimental Animals (Protocol CICUAE/124-2017; Approval date September 12, 2017) of the National Institute of Agricultural Technology (INTA) of Argentina.

### Study location, period, experimental design and animal procedures

The experiment was conducted at the Experimental Dairy Centre of the Balcarce Agricultural Experimental Station of INTA, Argentina (37°45’37”S; 58°17’55”W), during the period from October 20 to December 4, 2017. Eighteen calves (seven heifers and 11 steers) of 8.1±0.5 months of age (mean±standard deviation) and with 214±13.5 kg live weight were used. The calves were considered clinically healthy based on physical examination and blood sample results (biochemical and hematological parameters). During the study period, daily physical examination of the animals was performed, and potential lack of appetite, mucosal color or other abnormal signs were recorded. As a precautionary protocol, against intoxication of NO_3_^-^, a solution of methylene blue was prepared for emergency use, at a dose of 15 mg/kg of body weight (intravenous administration).

The animals were randomly allocated to either a control diet (CD; including five steers and four heifers) or a nitrate diet (ND; including six steers and three heifers). The CD group received a total mix ration (% of dry matter [DM]) of corn ground, soybean meal, premix, and urea (79.6%), and grass hay (20.4%). In turn, the ND group received CD (98.5%) plus 1.5% of NO_3_^-^ (as calcium NO_3_^-^, YaraLiva Calcinit^®^, Yara Argentina S.A.) (Table-1). The intermediate level of NO_3_^-^ inclusion in the diet was selected for this study because it was previously used to mitigate enteric CH_4_ emissions in Holstein cattle without compromising animal health [10,13].

**Table-1:** Dietary ingredients (% of DM) and nutritional composition of experimental diets (% of DM).

Variable	CD	ND
Ingredients		
Grass hay	20.4	20.4
Ground corn	69.4	68.3
Soybean expeller	8.0	8.4
Urea	0.8	0.2
Calcium nitrate*	0.0	1.5
Premix¥	1.1	1.1
Total	100	100
Composition		
Dry matter (% of FM)	90.4	90.5
Organic matter (% of DM)	94.5	94.5
Crude protein (% of DM)	12.4	12.2
Neutral detergent fiber (% of DM)	25.9	25.8
Starch (% of DM)	48.3	47.6
GE (MJ/kg of DM)	21.3	21.3

DM=Dry matter, FM=Fresh matter, GE=Gross energy, CD=Control diet, ND=Nitrate diet.

* Calcium ammonium nitrate, 5Ca (NO_3_)_2_•NH_4_NO_3_•10H_2_O; 75% NO_3_ on dry basis; estimated composition 11.3 g NO3^-^ /kg DM for nitrate treatment.

¥ Composition of Premix (per kg of premix): Calcium 23%, Sodium 8%, Phosphorus 1%, Magnesium 3,1%, Vitamin A 150000 UI, Vitamin D3 15000 UI, Vitamin E 150 UI, Iron 960 ppm, Magnesium 900 ppm, Zinc 900 ppm, Copper 150 ppm, Iodine 24 ppm, Cobalt 15 ppm, Selenium 6 ppm

To reduce the risks of toxicity, the amount of NO_3_^-^ was gradually increased (Table-2). The animals were fed *ad libitum* twice a day (08:00 AM and 4:00 PM) in individual pens (36 m^2^) provided with individual feeders and shared drinking troughs. The trial included 30 days of adaptation period to the diet and handling, followed by a 15 days measurement period (from day 31 to day 45).

**Table-2:** Scheme of progressive adaptation to a diet with NO_3_– inclusion.

Phase	1	2	3	4	5	6
Day	1-6	7-12	13-18	19-24	25-30	31-45
Calcium nitrate* (%)	20	40	60	80	100	100
g/animal/day	24.6	49.2	73.8	98.4	123	123

* Percentage of NO_3_– inclusion in each phase was according to the total intake on day 25 (15 g of NO_3_–/kg of DM)

### Blood sampling and analysis

Blood samples were taken only from the ND group. For blood gas (MetHb, oxyhemoglobin [O_2_Hb], carboxyhemoglobin [COHb], and deoxyhemoglobin [HHb]), hematocrit, and glucose monitoring, the sampling was performed 3 h post-feeding on days-1 (control day), 1, 7, 13, 19, and 25, by jugular vein puncture using Vacuette^®^ tubes with lithium heparin (Greiner Bio-One GmbH – Germany), and placed on ice directly after sampling. The analytes were determined using the Cobas-b221 blood gas system (Roche Diagnostics, USA).

In addition, the serum concentration of urea, albumin, aspartate aminotransferase (AST), alanine aminotransferase (ALT), gamma-glutamyl transpeptidase (GGT), and retinol was monitored, to examine liver function. Blood sampling on days-1 (control day), 7, and 25 were transferred into tubes with clot activator and gel separator (Greiner Bio-One GmbH - Germany). After clotting, serum was separated by low-speed centrifugation (3500× *g*) for 15 min at 4°C and stored at −20°C until analysis. The concentrations of urea and albumin were determined by the enzymatic method UV-glutamate dehydrogenase and colorimetrically with bromine cresol-sulfonephthalein, respectively [14], while AST and ALT and GGT were determined using an automatic biochemistry analyzer [15,16]. Retinol was determined as an indicator of Vitamin A by high-performance liquid chromatography.

### Evaluation of DM intake (DMI), LWG, and diets analysis

DMI was calculated as the difference between the daily offered and residual feed. Only measures of DMI from day 31 to day 45 (post-adaptation period) were considered for the analysis of the data. The results were expressed in kilograms of DMI/day. The LWG was determined as the difference between the final and initial weight during 45 days of evaluation and was expressed in kilograms of LWG/day.

The ingredients of the diets were dried in a forced-air oven at 55°C and milled to pass a 1-mm screen. DM analysis by oven drying (105°C) and ash by incineration at 550°C for 4 h were determined, according to AOAC [17]. Total nitrogen content was determined by combustion type auto-analyzer (Leco FP-2000, Leco Corp., St. Joseph, MI). In addition, we assessed neutral detergent fiber in a fiber analyzer ANKOM^®^ 220 (ANKOM Technology, Macedon NY-USA) [18], and starch was analyzed by an enzymatic method [19].

### Statistical analysis

The results of DMI and LWG were analyzed with PROC MIXED SAS software version 13.1 (SAS Institute Inc., Cary NC, USA 2013) [20] with treatment as fixed effect and animals as random effect according to the model *Y_ij_*=*µ+Treat_i_+Anim_j_* *(Treat)+e_ij_*, where: Y*_ij_*=response variable; *µ*=general mean of the experiment; *Treat_i_*=*Treatment*, CD versus ND (*i*=2); *Anim_j_* (*Treat*)=animals within the treatment (*j*=18); *e_ij_*=experimental error.

Urea, albumin, retinol, AST, ALT, and GGT data were analyzed with the time factor as a repeated measure using PROC GLM of the SAS version 13.1 (SAS Institute Inc., Cary NC, USA 2013) [20], according to the following model: *Y_ij_*=*µ*+*Anim_i_*+*Time_j_*+*e_ij_*, where: *Y_ij_*=response variable; *µ*=general mean of the experiment; *Anim_i_*=animals (*i*=9); *Time_j_*=time factor: sampling day (*j*=6 or 3); *e_ij_*=experimental error, in turn, followed by Dunnett’s multiple comparison tests.

The data that did not meet the assumption of normality and homogeneity of variance, such as MetHb, O_2_Hb, COHb, and HHb, glucose and hematocrit were analyzed using the Friedman test and a comparison between median was performed using Wilcoxon signed-rank test in R software version 3.6.1 [21]. Differences among mean and median were considered significant when p<0.05. In addition, Spearman’s correlation analysis was used to evaluate the association between variables using the *corrplot* function in R.

## Results

### Effect on DMI and LWG

DMI and LWG did not differ among dietary treatments in Holstein calves (p=0.117 and p=0.439, respectively; Table-3). Likewise, the initial and final weight of the calves did not differ significantly among dietary treatments (p=0.960 and p=0.832, respectively).

**Table-3:** Dry matter intake and live weight gain in Holstein calves fed with a control diet (n=9) and nitrate diet (n=9).

Parameters	Diets		SEM	p-value

	CD	ND		
Dry matter intake (kg/day)	8.8	8.2	0.24	0.117
Initial weight (kg)	214	214	4.76	0.960
Final weight (kg)	268	266	5.81	0.832
Live weight gain (kg/day)	1.2	1.1	0.10	0.439

SEM=Standard error of the mean, CD=Control diet, ND=Nitrate diet

### Effect on hematological, biochemical, and blood gases parameters

**Figure-1:**
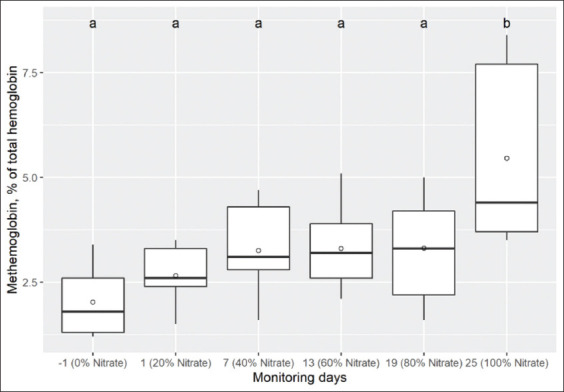
Box and whisker plots showing levels of methemoglobin (% of total hemoglobin) in blood of nine Holstein calves measured on day-1, 1, 7, 13, 19, and 25 with 0, 20, 40, 60, 80, and 100% of total NO_3_^-^ in the diet, respectively. The median is indicated by the middle line, the mean is indicated by the symbol (○), and the 75^th^ and 25^th^ percentiles by the upper and lower edges of the boxes. The whiskers show the 95% confidence interval. Comparison of medians, box and whisker with different letters above (“a” or “b”) differs (p<0.05) from day-1 (range test signed by Wilcoxon).

An incremental effect was observed for MetHb (Figure-1), where levels increased numerically until day 19, though not significantly compared to day-1. In contrast, on day 25, there was a significant increase compared to day-1 (p<0.001). Moreover, an oppositive effect was observed for the O_2_Hb level, because it decreased according to NO_3_^-^ concentrations in the diet, although there was a significant decrease only on day 25 compared to day-1 (p<0.001; Table-4). In turn, the COHb values decreased significantly on days 1, 13, 19, and 25 with respect to day-1 (p=0.003), but statistically significant differences were not found between day 7 and day-1. Conversely, HHb increased significantly from day 1 to day 13 compared to day-1, and then there was a slight decrease toward day 25, but remained higher than day-1 (p=0.005).

**Table-4:** Effect of a progressive inclusion of NO_3_– in the diet on blood gases (%) and hematocrit (%) levels in Holstein calves (n=9).

Parameters	Monitoring days (medians±IQR)*						p-value	Reference values

	Day-1	Day 1	Day 7	Day 13	Day 19	Day 25		
O_2_Hb	96±1.8^a^	94±1.2^a^	95±2.9^a^	92±3.7^a^	93±3.4^a^	90±3.3^b^	0.001	N/A
COHb	1.5±2.4^a^	0.4±0.2^a^	0.7±0.3^a^	0.4±0.2^b^	0.3±0.3^b^	0.4±1.1^b^	0.003	N/A
HHb	0.8±0.1^a^	2.7±1.1^b^	2.1±1.7^b^	5.5±4.0^b^	3.5±2.5^b^	1.7±3.9^b^	0.005	N/A
Hematocrit	32±1.7^a^	30±0.8^b^	29±1.8^b^	29±0.8^b^	29±1.3^b^	29±0.5^b^	0.001	30-36% Kahn and Line [38]

O_2_Hb=Oxyhemoglobin, COHb=Carboxyhemoglobin, HHb=Deoxyhemoglobin. IQR=Interquartile range. N/A=Not applicable.

* Day-1=Without NO_3_– in the diet (control day); Day 1=With 20% of total NO_3_– in the diet; Day 7=With 40% of total NO_3_– in the diet; Day 13=With 60% of total NO_3_– in the diet; Day 19=With 80% of total NO_3_– in the diet; Day 25=With 100% NO_3_– in the diet. ^a,b^Medians within a row with different superscripts differ (p<0.05) from Day-1 (Wilcoxon signed-rank test)

**Figure-2:**
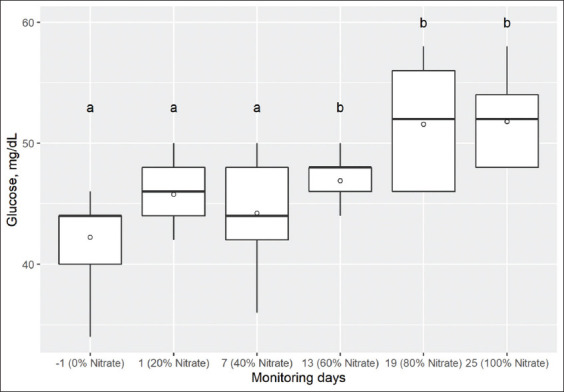
Box and whisker plots showing glucose concentrations (mg/dL) in blood of nine Holstein calves measured on day-1, 1, 7, 13, 19, and 25 with 0, 20, 40, 60, 80, and 100% of total NO_3_^-^ in the diet, respectively. The median is indicated by the middle line, the mean is indicated by the symbol (○), and the 75^th^ and 25^th^ percentiles by the upper and lower edges of the boxes. The whiskers show the 95% confidence interval. Comparison of medians, box and whisker with different letters above (“a” or “b”) differs (p<0.05) from day-1 (range test signed by Wilcoxon). Reference values: 42-75 mg/dL Kahn and Line [38].

The hematocrit was reduced according to NO_3_^-^ concentrations in the diet (p=0.001). This reduction was not associated with the hemolysis of blood samples because they were verified during laboratory analyses. In turn, glucose concentrations increased with NO_3_^-^ inclusion (p=0.001), being most evident on days 13, 19, and 25, which corresponded to 60, 80, and 100% of NO_3_^-^ inclusion (Figure-2).

**Figure-3:**
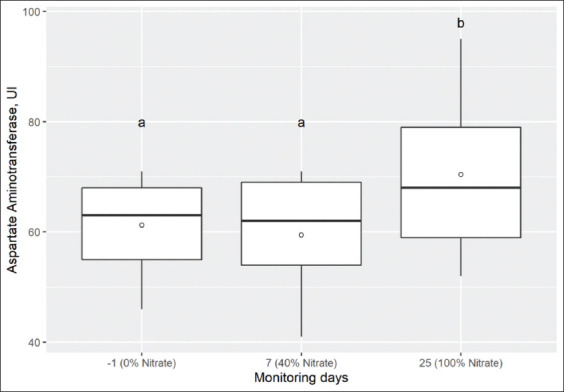
Box and whisker plots showing aspartate aminotransferase activity (UI) in serum of nine Holstein calves measured on day-1, 7, and 25 with 0, 40, and 100% of total NO_3_^-^ in the diet. The median is indicated by the middle line, the mean is indicated by the symbol (○), and the 75^th^ and 25^th^ percentiles by the upper and lower edges of the boxes. The whiskers show the 95% confidence interval. Comparison of means, box and whisker with different letters above (“a” or “b”) differs (p<0.05) from day-1 (Dunnett’s test). Reference values: 78-132 UI, Kaneko et al [37].

**Figure-4:**
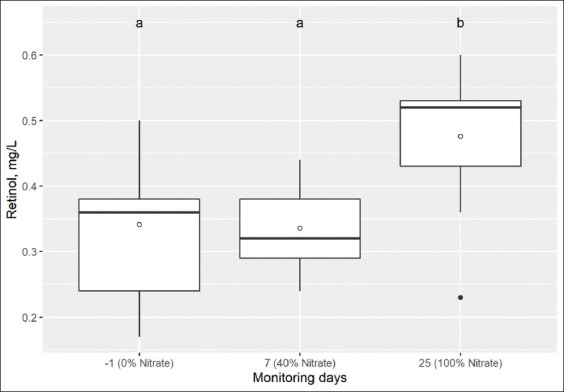
Box and whisker plots showing retinol concentrations (mg/L) in serum of 9 Holstein calves measured on day-1, 7, and 25 with 0, 40, and 100% of total NO_3_^-^ in the diet. The median is indicated by the middle line, the mean is indicated by the symbol (○), and the 75^th^ and 25^th^ percentiles by the upper and lower edges of the boxes. The whiskers show the 95% confidence interval. Comparison of means, box and whisker with different letters above (“a” or “b”) differs (p<0.05) from day-1 (Dunnett’s test). Reference values: Higher than 0.20 mg/L, Bouda [36].

On the other hand, the changes of AST activity (Figure-3) and retinol concentrations (Figure-4) on day 7 (corresponding to 40% of total NO_3_^-^) in comparison to day-1 were not different. However, on day 25, there was a significant increase with respect to day-1 (p=0.004 and p=0.025, respectively). Similarly, there was a significant increase in urea concentrations from day 7 to day 25 compared to day-1 (p=0.001). However, the levels of NO_3_^-^ inclusion in the diet did not modify albumin concentrations, and ALT and GGT activity (p=0.387, p=0.673, and p=0.779, respectively) in Holstein calves (Table-5).

**Table-5:** Effect of a progressive inclusion of NO_3_– in the diet on biochemical parameters and liver enzymes in blood serum of Holstein calves (n=9).

Parameters	Monitoring days (means)*			SEM	p-value	Reference values

	Day-1	Day 7	Day 25			
Urea (mg/dL)	14^a^	23^b^	21^b^	0.93	0.001	10-25 Kahn and Line [38]
Albumin (g/L)	38	39	37	0.09	0.387	25-38 Kahn and Line [38]
ALT (UI)	16	16	17	0.97	0.673	11-40 Kaneko *et al* [37]
GGT (UI)	22	21	22	0.83	0.779	6.1-17.4 Kaneko *et al* [37]

ALT=Alanine aminotransferase, GGT=Gamma-glutamyl transpeptidase. SEM=Standard error of the mean.

* Day-1=Without NO_3_–in the diet; Day 7=With 40% of total NO_3_– in the diet; day 25=With 100% NO_3_^–^in the diet. ^a,b^Means within a row with different superscripts differ (p<0.05) from Day-1 (Dunnett’s test)

### Correlation analysis of DMI, LWG, and blood parameters

**Figure-5:**
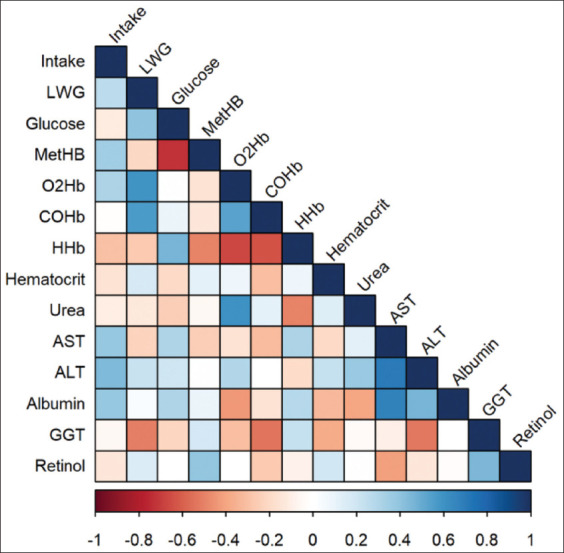
Correlogram of Spearman’s correlation analysis between dry matter intake, LWG, and blood parameters (Glucose; MetHB=Methemoglobin; O_2_Hb=Oxyhemoglobin; COHb=Carboxyhemoglobin; HHb=Deoxyhemoglobin; Hematocrit; Urea; AST=Aspartate Aminotransferase; ALT=Alanine aminotransferase; Albumin; GGT=Gamma-glutamyl transpeptidase and retinol). The data of the blood parameters correspond to the 25^th^ day of adaptation to the diet (100% of NO_3_^-^). LWG=Live weight gain.

Correlation analyses were performed with the hematological, biochemical, and blood gases variables corresponding to day 25 (Figure-5). DMI was positively associated with the level of MetHb (r=0.34), O_2_Hb (r=0.32), AST (r=0.38), ALT (r=0.44), and albumin (r=0.38). In contrast, it was negatively associated and in less degree with the concentration of glucose (r=−0.12), HHb (r=−0.30), hematocrit (r=−0.15), urea (r=−0.10), and retinol (r=−0.13). LWG was positively associated with glucose concentration (r=0.41), O_2_Hb (r=0.6), and COHb (r=0.57), while, negatively with the concentration of MetHb (r=−0.2), HHb (r=−0.27), Urea (r=−0.13), AST (r=−0.23), and GGT (r=−0.5). MetHb was negatively associated with glucose concentration (r=−0.73), and less so with O_2_Hb (r=−0.15), COHb (r=−0.13), HHb (r=−0.49), and AST (r=−0.25), but positively with retinol (r=0.39). Urea concentration was positively associated with O_2_Hb (r=0.6) and ALT (r=0.38), and negatively with HHb (r=−0.31) and albumin (r=−0.39). AST activity was positively correlated with ALT activity (r=0.7) and albumin concentration (r=0.68), and negatively with retinol (r=−0.41). GGT activity was negatively associated with ALT (r=−0.53) and COHb (r=−0.54), and positively with retinol (r=0.45).

## Discussion

The physiological response to NO_3_^-^ in animals was variable because the level of NO_3_^-^ toxicity depends on several factors: Dietary NO_3_^-^ dose levels, the rate of NO_3_^-^ intake, an incomplete reduction of NO_3_^-^ and NO2_-_ to NH_3_ in the rumen, and a low rate of rumen content passage, which results in higher retention of NO_3_^-^ or NO2_-_ in the rumen [22]. Thus, in this study, it was possible to control the majority of these risk factors by the progressive inclusion of NO_3_^-^ in the diet because it allowed the DMI and LWG not to differ between dietary treatments, despite observing a 7% numerical reduction in the DMI in the DN, which could be attributed to the organoleptic characteristics of NO_3_^-^ [5]. Similar results were found in the previous studies [23,24].

A linear relationship between levels of blood MetHb and dietary NO_3_^-^ was observed in a meta-analysis study [1]. In addition, Newbold *et al*. [25] showed that CH_4_ emissions decreased linearly with increasing dietary NO_3_^-^ level, but the risk of poisoning also increased with daily doses >2.4 g of NO_3_^-^/kg of DM. However, when NO_3_^-^ was used in intermediate doses (13-21 g of NO_3_^-^/kg of DM/day) and adequate dietary adaptation studies showed a 14-25% decrease in enteric CH_4_ emissions without affecting animal performance and animal health [10,11,26,27]. In this study, blood MetHb did not exceed the upper tolerance limits for cattle (<10% of total hemoglobin) when fed daily with 15 g of NO_3_^-^/kg of DM, but we noted that the individual response was variable (CV=38.4%). Moreover, the percentage of MetHb was positively associated with DMI, and negatively with LWG, HHb, O_2_Hb, and COHb. Furthermore, although NO_3_^-^ did not affect LWG, the numerical difference (−100 g) could be explained partially by the negative association between these variables. In the previous studies in beef and dairy cattle under a system of progressive adaptation to dietary NO_3_^-^ reported levels <6% of blood MetHb [11,24]. Similar results were found using encapsulated NO_3_^-^ [7].

The increase in serum urea concentrations according to the increase in dietary NO_3_^-^ was expected, since NO_3_^-^ in the rumen is reduced to NH_3_ by NO_3_^-^-reducing microorganisms. Therefore, these increases can be attributed to a higher concentration of rumen NH_3_, as it is absorbed and converted to urea in the liver, then excreted through the urine [28]. Furthermore, the increased concentration of glucose is probably related to high urea concentration. This mechanism in ruminants is well documented and has been attributed either to a lower release of pancreatic insulin [29] or to increased glucose production in the liver [30]. However, some authors hypothesized the beneficial effects of using NO_3_^-^ and NO2_-_ as precursors of nitric oxide, at blood and tissue level, on glucose uptake and increased insulin sensitivity in humans and rats [31,32], but not confirmed yet in ruminants [33].

Overall, we can affirm that the progressive inclusion of 123 g of NO_3_^-^/animal/day, caused an animal metabolic adjustment, due to a reduction of available oxygen caused by the increase of blood MetHb induced a higher glucose concentration and a reduction of hematocrit because aerobic metabolism at cellular and tissue level was affected by the reduction of oxygen availability [34]. However, animals with higher MetHb levels did not always induce higher glucose concentration, showing a negative correlation between both variables. The reason for these findings remains unclear.

Recently, González Delgado *et al*. [34] studied the acute effects of NO_3_^-^ poisoning in Wistar rats. The authors observed an increase in glucose, cholesterol, triglycerides, LDH, AST, and ALT that associated with changes in liver metabolism caused by liver damage. Moreover, other study reported that levels of LDH, AST, and ALT were increased under the chronic condition of NO_3_^-^ exposure in pregnant cows [35]. However, in this study, no significant increase in liver enzymes was observed after NO_3_^-^ inclusion. These different results are probably due to NO_3_^-^ exposure time, physiological status of the animal, animal species, and dose levels of NO_3_^-^, time of adaptation to NO_3_^-^, among other factors.

We can confirm the hypothesis that the progressive inclusion of NO_3_^-^ allows an effective adaptation of the NO_3_^-^-reducing ruminal microbiota, without reaching toxicity levels for the animal, nor causing changes in animal performance. There were no changes in DMI, LWG, nor ALT and GGT activity, or albumin concentration by NO_3_^-^ inclusion in the diet, except for MetHb, urea, glucose, AST, and retinol concentrations that were significantly increased. However, these increases did not exceed the reference values of clinically healthy cattle [36-38].

## Conclusion

This study confirmed that the progressive inclusion of 123 g of NO_3_^-^/animal/day in the diet could provide safe supplementation for Holstein calves without affecting DMI and LWG. In turn, a dose-response effect of the MetHb, glucose, urea, AST, and retinol was observed, but these values did not exceed reference values. These results highlighted the importance of using a scheme of progressive inclusion of NO_3_^-^ in the diet of calves to reduce the risks of NO_3_^-^ toxicity.

## Authors’ Contributions

AO designed and performed the experiments, analyzed, and wrote the manuscript. GM, GD, and FS contributed in handling the animals, sample collection, and analyzed the data. MDT, JG, CF, and SLC contributed reagents/materials and analyzed the data. AC designed the experiment and analyzed the data. MEC conceived and designed the experiments, analyzed the data, and reviewed drafts of the paper. All authors have checked and approved the final version of the manuscript.

## References

[1] Lee C, Beauchemin K.A (2014). A review of feeding supplementary nitrate to ruminant animals:Nitrate toxicity, methane emissions, and production performance. Can. J. Anim. Sci.

[2] Latham E.A, Anderson R.C, Pinchak W.E, Nisbet D.J (2016). Insights on alterations to the rumen ecosystem by nitrate and nitrocompounds. Front. Microbiol.

[3] Kemp A, Geurink J.H, Haalstra R.T, Malestein A (1977). Nitrate poisoning in cattle. 2. Changes in nitrite in rumen fluid and methemoglobin formation in blood after high nitrate intake. Netherlands J. Agric. Sci.

[4] Yaremcio B (1991). Nitrate poisoning and feeding nitrate feeds to livestock. [Online].

[5] Bruning-Fann C.S, Kaneene J.B (1993). The effects of nitrate, nitrite, and N-nitroso compounds on animal health. Vet. Hum. Toxicol.

[6] Beauchemin K.A, Ungerfeld E.M, Eckard R.J, Wang M (2020). Review:Fifty years of research on rumen methanogenesis: Lessons learned and future challenges for mitigation. Animal.

[7] Lee C, Araujo R.C, Koenig K.M, Beauchemin K.A (2015). Effects of encapsulated nitrate on eating behavior, rumen fermentation, and blood profile of beef heifers fed restrictively or ad libitum. J. Anim. Sci.

[8] Zhang X, Medrano R.F, Wang M, Beauchemin K.A, Ma Z, Wang R, Wen J, Bernad L.A, Tan Z (2019). Effects of urea plus nitrate pretreated rice straw and corn oil supplementation on fiber digestibility, nitrogen balance, rumen fermentation, microbiota and methane emissions in goats. J. Anim. Sci. Biotechnol.

[9] Guyader J, Doreau M, Morgavi D.P, Gerard C, Loncke C, Martin C (2016). Long-term effect of linseed plus nitrate fed to dairy cows on enteric methane emission and nitrate and nitrite residuals in milk. Animal.

[10] van Zijderveld S.M, Gerritis W.J.J, Dijkstra J, Newbold J.R, Hulshof R.B.A, Perdok H.B (2011). Persistency of methane mitigation by dietary nitrate supplementation in dairy cows. J. Dairy Sci.

[11] Olijhoek D.W, Hellwing A.L.F, Brask M, Weisbjerg M.R, Højberg O, Larsen M.K, Dijkstra J, Erlandsen E.J, Lund P (2016). Effect of dietary nitrate level on enteric methane production, hydrogen emission, rumen fermentation, and nutrient digestibility in dairy cows. J. Dairy Sci.

[12] Wang R, Wang M, Ungerfeld E.M, Zhang X.M, Long D.L, Mao H.X, Deng J.P, Bannik A, Tan Z.L (2018). Nitrate improves ammonia incorporation into rumen microbial protein in lactating dairy cows fed a low-protein diet. J. Dairy Sci.

[13] Leng R.A, Preston T.R, Inthapanya S (2012). Biochar reduces enteric methane and improves growth and feed conversion in local yellow cattle fed cassava root chips and fresh cassava foliage. Livest. Res. Rural Dev.

[14] Doumas B.T, Ard Watson W, Biggs H.G (1971). Albumin standards and the measurement of serum albumin with bromcresol green. Clin. Chim. Acta.

[15] Szasz G, Bergmeyer H.U (1974). Gamma-glutamyltranspeptidase. Methoden, der Enzymatis Chen Analyse.

[16] Thefeld W, Hoffmeister H, Busch E.W, Koller P.U, Vollmar J (1974). Referenzwerte für die bestimmungen der transaminasen GOT und GPT sowie der alkalischen phosphatase im serum mit optimierten standard methodsen. Dtsch. Med. Wochenschr.

[17] AOAC (1990). Official Methods of Analysis.

[18] Van Soest P.J, Robertson J.B, Lewis B.A (1991). Methods for dietary fiber, neutral detergent fiber, and nonstarch polysaccharides in relation to animal nutrition. J. Dairy Sci.

[19] MacRae J.C, Armstrong D.G (1968). Enzyme method for determination of a-linked glucose polymers in biological materials. J. Sci. Food Agric.

[20] SAS (2013). SAS/STAT®13.1 User's Guide.

[21] R Core Team (2019). R:A Language and Environment for Statistical Computing.

[22] Leng R.A (2008). The Potential of Feeding Nitrate to Reduce Enteric Methane Production in Ruminants Report to Department of Climate Change, Commonwealth Government, Canberra.

[23] Hulshof R.B.A, Berndt A, Gerrits W.J.J, Dijkstra J, van Zijderveld S.M, Newbold J.R, Perdok H.B (2012). Dietary nitrate supplementation reduces methane emission in beef cattle fed sugarcane-based diets. J. Anim. Sci.

[24] Doreau M, Arbre M, Popova M, Rochette Y, Martin C (2017). Linseed plus nitrate in the diet for fattening bulls:Effects on methane emission, animal health and residues in offal. Animal.

[25] Newbold J.R, van Zijderveld S.M, Hulshof R.B.A, Fokkimk W.B, Leng R.A, Terencio P, Powers W.J, van Adrichem P.S.J, Paton N.D, Perdok H.B (2014). The effect of incremental levels of dietary nitrate on methane emissions in Holstein steers and performance in Nelore bulls. J. Anim. Sci.

[26] Lund P, Dahl R, Yang H.J, Hellwing A.L.F, Cao B.B, Weisbjerg M.R (2014). The acute effect of addition of nitrate on *in vitro* and *in vivo* methane emission in dairy cows. Anim. Prod. Sci.

[27] Klop G, Hatew B, Bannik A, Dijkstra J (2016). Feeding nitrate and docosahexaenoic acid affects enteric methane production and milk fatty acid composition in lactating dairy cows. J. Dairy Sci.

[28] Abdoun K, Stumpff F, Martens H (2007). Ammonia and urea transport across the rumen epithelium:A review. Anim. Health Res. Rev.

[29] Fernandez J.M, Croom W.J, Tate L.P, Johnson A.D (1990). Subclinical ammonia toxicity in steers:Effects on hepatic and portal-drained visceral flux of metabolites and regulatory hormones. J. Anim. Sci.

[30] Huntington G.B, Harmon D.L, Kristensen N.B, Hanson K.C, Spears J.W (2006). Effects of a slow-release urea source on absorption of ammonia and endogenous production of urea by cattle. Anim. Feed Sci. Technol.

[31] Lundberg J.O, Weitzberg E, Gladwin M.T (2008). The nitrate-nitrite-nitric oxide pathway in physiology and therapeutics. Nat. Rev. Drug Discov.

[32] Khalifi S, Rahimipour A, Jeddi S, Ghambari M, Kazerouni F, Ghasemi A (2015). Dietary nitrate improves glucose tolerance and lipid profile in an animal model of hyperglycemia. Nitric. Oxide.

[33] Villar M.L, Godwin I.R, Hegarty R.S, Dobos R.C, Smith K.A, Clay J.W, Nolan J.V (2019). The effects of dietary nitrate on plasma glucose and insulin sensitivity in sheep. J. Anim. Physiol. Anim. Nutr.

[34] González Delgado M.F, González Zamora A, Gonsebatt M.E, Meza Mata E, Garcia Vargas G.G, Calleros Rincon E.Y, Pérez Morales R (2018). Subacute intoxication with sodium nitrate induces hematological and biochemical alterations and liver injury in male Wistar rats. Ecotoxicol. Environ. Saf.

[35] Sezer K, Albay M.K, Ozmen O, Haligur M, Sahinduran S, Mor F, Koker A (2011). Hematological, biochemical and thyroid gland investigations in pregnant cows and in calves chronically intoxicated with nitrate. Rev. Med. Vet.

[36] Bouda J (1984). Biochemical and hematological values in calves and their significance for health control. Acta Vet. Brno.

[37] Kaneko J.J, Harvey J.W, Bruss M.L (2008). Clinical Biochemistry of Domestic Animals 6^th^ ed.

[38] Kahn C.M, Line S (2010). Reference guides. The Merck Veterinary Manual.

